# Risk Factors Associated With Peripartum Suicide Attempts in Japan

**DOI:** 10.1001/jamanetworkopen.2022.50661

**Published:** 2023-01-12

**Authors:** Tetsuya Akaishi, Kunio Tarasawa, Kiyohide Fushimi, Hirotaka Hamada, Masatoshi Saito, Natsuko Kobayashi, Saya Kikuchi, Hiroaki Tomita, Tadashi Ishii, Kenji Fujimori, Nobuo Yaegashi

**Affiliations:** 1 Department of Education and Support for Regional Medicine, Tohoku University Hospital, Sendai, Japan; 2 Department of Health Administration and Policy, Tohoku University Graduate School of Medicine, Sendai, Japan; 3 Department of Health Policy and Informatics, Tokyo Medical and Dental University Graduate School of Medical and Dental Sciences, Tokyo, Japan; 4 Department of Obstetrics and Gynecology, Tohoku University Graduate School of Medicine, Sendai, Japan; 5 Department of Psychiatry, Tohoku University Graduate School of Medicine, Sendai, Japan

## Abstract

**Importance:**

Peripartum suicide attempt is a major psychiatric complication associated with pregnancy, but the risk factors remain largely uncertain.

**Objective:**

To identify the demographic characteristics and predisposing risks for peripartum suicide attempts and postpartum depression.

**Design, Setting, and Participants:**

This cohort study used retrospective data on pregnant women who delivered children between April 1, 2016, and March 31, 2021, at 712 hospitals in Japan. The nationwide Diagnosis Procedure Combination database was used.

**Exposures:**

Psychiatric and nonpsychiatric medical history, age, alcohol and tobacco use, and obstetric complications and procedures.

**Main Outcomes and Measures:**

Data on admissions for prepartum suicide attempt and delivery during the same hospital stay and readmissions for depression or suicide attempt within 1 year post partum were collected. Comparisons of prevalence of each study variable were performed, and multivariable logistic regression analyses were used to determine risk factors.

**Results:**

From a total of 39 908 649 hospitalization episodes, 804 617 cumulative pregnant women (median [IQR] age at childbirth, 33 [29-36] years) who delivered at the enrolled hospitals were identified, including 1202 who were admitted for suicide attempt and delivery during the same hospital stay and 111 readmitted for suicide attempt within 1 year post partum. Risk factors associated with prepartum suicide attempts included younger age (adjusted odds ratio [aOR], 0.99; 95% CI, 0.98-1.00) and histories of personality disorder (aOR, 10.81; 95% CI, 5.70-20.49), depression (aOR, 3.97; 95% CI, 2.35-6.70), schizophrenia (aOR, 2.89; 95% CI, 1.52-5.50), and adjustment disorder (aOR, 2.66; 95% CI, 1.07-6.58). Risk factors associated with postpartum suicide attempts included younger age (aOR, 0.96; 95% CI, 0.93-1.00), heavy tobacco use (aOR, 23.09; 95% CI, 5.46-97.62), and histories of alcohol use disorder (aOR, 163.54; 95% CI, 28.30-944.95), personality disorder (aOR, 10.28; 95% CI, 3.29-32.10), anxiety disorders (aOR, 8.13; 95% CI, 2.88-22.98), depression (aOR, 7.27; 95% CI, 2.95-17.91), schizophrenia (aOR, 5.77; 95% CI, 2.17-15.38), bipolar disorder (aOR, 3.98; 95% CI, 1.36-11.67), and insomnia (aOR, 3.17; 95% CI, 1.30-7.78). On sensitivity analysis, risk factors associated with postpartum depression after excluding those with prenatal depression included histories of personality disorder, adjustment disorder, bipolar disorder, insomnia, and anxiety disorders.

**Conclusions and Relevance:**

The findings of this cohort study suggest that histories of smoking and prenatal psychiatric disorders are potential risk factors for peripartum suicide attempts and may require additional treatment and prevention interventions.

## Key Points

**Question:**

Which aspects of psychiatric and nonpsychiatric medical histories are associated with the risk of peripartum suicide attempts?

**Findings:**

In this cohort study of 804 617 pregnant individuals in Japan, risk factors associated with peripartum suicide attempts were prenatal history of alcohol use disorder, anxiety disorders, personality disorder, schizophrenia, depression, being a heavy smoker, and younger age. These factors were significant for readmission for suicide attempt within 1 year post partum.

**Meaning:**

These findings suggest that mothers with heavy alcohol and/or tobacco use, anxiety disorders, personality disorder, schizophrenia, and depression should be observed closely during and after pregnancy to prevent a suicide attempt, especially in those who are younger.

## Teaser

This cohort study uses national hospital data to describe the risk factors associated with peripartum suicide attempts in pregnant individuals in Japan.

## Introduction

Childbirth is a major life event, with long-term physical and mental impacts.^1^ Approximately one-half of individuals who give birth experience some form of emotional instability within the first month post partum,^2,3^ most of which is self-remitting and does not usually require psychiatric treatment. In contrast, postpartum depression or suicide attempt is a psychiatric complication associated with childbirth that may harm both mother and child and requires careful follow-up and interventions.^4,5,6^ Up to 10% of pregnant individuals will experience some form of mental condition,^7,8^ some of which can have a lethal outcome.^9,10,11,12^ As maternal death due to critical obstetric hemorrhage has decreased worldwide, possibly through improvements in medical practice, equipment, and blood supply systems,^13,14,15,16^ the maternal death rate by suicide has increased. Suicidal death is currently considered to account for 5% to 20% of maternal mortality in developed countries,^17,18,19^ but the exact risks of peripartum suicide attempts remain largely unknown. Research into this topic through a large cohort is an urgent public health need. Therefore, the aim of this study is to clarify the features and risks of hospitalization for prepartum or postnatal suicide attempts.

## Methods

This cohort study was approved by the institutional review boards of Tokyo Medical and Dental University (approval No. M2000-788) and Tohoku University Graduate School of Medicine (approval No. 2022-1-441). The review boards waived the requirement for informed consent because the patient data were anonymous. The study was performed in accordance with the Strengthening the Reporting of Observational Studies in Epidemiology (STROBE) reporting guideline.^20^

### Data Source

We used the Japanese Diagnosis Procedure Combination (DPC) database, which contains data on inpatient care collected from more than 7 million people treated at hospitals annually. The DPC data cover approximately 70% of all hospitalization episodes annually in Japan. Anonymized data include diagnosed disease name and type according to the *International Statistical Classification of Diseases and Related Health Problems, Tenth Revision* (*ICD-10*)^21^; date registered by the participating hospital; and other comprehensive medical data, including age, dates of hospital admission and discharge, type of admission (planned or unplanned), summary on admission, outcome at hospital discharge, and discharge destination. Racial and ethnic data were not collected or analyzed.

### Study Design

We initially selected all hospitalization episodes registered in the DPC database for women who had given birth between April 1, 2016, and March 31, 2021. A total of 1540 hospitals across Japan participated in the DPC system. Hospitalization episodes involving childbirth were defined as eligible for the present study, and patients were evaluated for the prevalence of factors associated with the psychiatric outcomes under study. The control group comprised hospitalization episodes without any of the evaluated psychiatric outcomes. A flow diagram of the study design is shown in the Figure.

**Figure.**
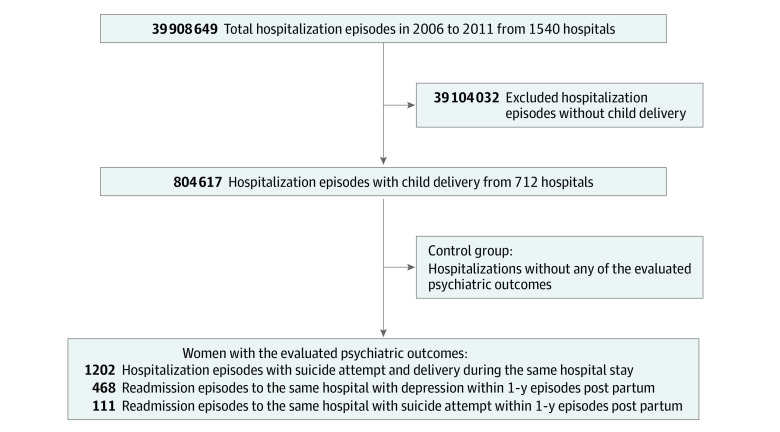
Flow Diagram of the Study Design Initially collected data regarding the overall hospitalization episodes in 2016-2021 are from 1540 hospitals in Japan that joined the nationwide Diagnostic Procedure Combination (DPC) program by the Japanese government, including all 82 university hospitals in the country. From these hospitalizations, DPC data of delivery hospitalizations from 712 hospitals were eligible for the present study. Measured outcomes were (1) admission for prepartum suicide attempt that was followed by delivery during the same hospital stay, (2) readmission to the same hospital of delivery for depression within 1 year post partum, and (3) readmission to the same hospital of delivery for suicide attempt within 1 year post partum.

### Psychiatric Outcomes

The psychiatric outcomes included (1) admission with prepartum suicide attempt and delivery during the same hospital stay, (2) readmission to the same hospital of delivery for depression within 1 year post partum, and (3) readmission to the same hospital of delivery for suicide attempt within 1 year post partum. Information on suicide attempts was based on the hospitalization summary on admission. Information on suicide attempts was registered when a suicide attempt was confirmed as a trigger for hospitalization by the patients themselves, their family members or acquaintances, or attending paramedics. Based on the current *ICD-10*, postpartum depression can be incorporated into 1 of the following disease classifications: major depressive episode (*ICD-10* code F32); recurrent depressive disorder (*ICD-10* code F33); or mental and behavioral disorders associated with the puerperium, or not elsewhere classified (*ICD-10* code F53). To distinguish self-remitting postpartum emotional instability from postpartum depression, patients given the *ICD-10* code F53 within the first week post partum were not considered to have postpartum depression. Furthermore, women with manic episode (*ICD-10* code F30) or bipolar affective disorder (*ICD-10* code F31) after childbirth were not regarded as having postpartum depression. Based on the current general agreement for the latest period to diagnose postpartum depression at approximately 12 months post partum,^22,23,24^ all episodes of readmission with depression or suicide attempt to the same hospital of delivery within 1 year post partum were considered as eligible outcomes.

### Potential Risk Factors

The following information was collected on admission to hospitals where childbirth occurred: age, body mass index (BMI) calculated as weight divided by height in meters squared, smoking history and Brinkman index, prenatal nonpsychiatric medical history, prenatal psychiatric history, and types of antidepressants or sedative drugs administered. The evaluated nonpsychiatric histories included diabetes, hypertension, dyslipidemia, Graves disease, Hashimoto thyroiditis, systemic lupus erythematosus, Sjögren syndrome, rheumatoid arthritis, epilepsy, multiple sclerosis, and long-term use of oral corticosteroids. The evaluated prenatal psychiatric histories included depression, schizophrenia, alcohol use disorder, personality disorder, insomnia, anxiety disorders, bipolar disorder, adjustment disorder, and prenatal suicide attempts. Types of antidepressants and sedative-hypnotics used were also evaluated. Sedative-hypnotics were categorized as benzodiazepines, non-benzodiazepine γ-aminobutyric acid receptor agonists (Z-drugs), and others. Peripartum obstetric procedures and comorbidities, including elective cesarean delivery (CD), emergency CD, vacuum extraction, threatened preterm labor, preterm labor, threatened miscarriage, miscarriage, stillbirth, postterm birth, gestational hypertension, and lactation mastitis, were also collected.

### Statistical Analysis

Comparison of the prevalence of each risk factor between patients with and without each outcome was performed using the χ^2^ or Fisher exact test. Two-sided *P* values and effect sizes with odds ratios (ORs) are reported. Comparisons of quantitative data were performed using the Mann-Whitney *U* (MWU) test. Following the initial bivariate analyses, multivariable binary logistic regression analyses were performed. Age on admission and prenatal psychiatric problems with *P* < .10 in the bivariate analyses were included as explanatory variables. All selected risk factors were simultaneously entered into the respective regression models. Multicollinearity among the risk factors was evaluated by calculating the variance inflation factor for each variable. To check for the robustness of the obtained statistical significances from the multivariable analysis, regardless of the prenatal history of each outcome, sensitivity analyses, after excluding patients with a prenatal history of each outcome, were further performed. For both the bivariate and multivariable analyses, the threshold for statistical significance was consistently set at 2-sided *P* < .05 based on the exploratory nature of the bivariate analyses.^25^ R, version 4.0.5 statistical software (R Foundation for Statistical Computing) was used for the analyses.

## Results

### Participants

From among 39 908 649 hospitalization episodes (1540 hospitals), 804 617 hospitalizations for delivery were identified from 712 hospitals. Deliveries included not only live births but also miscarriage (4813 deliveries) and stillbirths (289 deliveries). Patients’ median age at childbirth was 33 years (IQR, 29-36 years). Median BMI was 24.5 (IQR, 22.3-27.1). The patients’ nonpsychiatric medical histories are summarized in Table 1. Smoking history was confirmed in 69 940 of the 804 617 women (8.69%), including 562 (0.07%) heavy smokers with a Brinkman index of 600 or greater.

**Table 1.** Nonpsychiatric Medical and Obstetric History of Patients Readmitted for Postpartum Depression or Suicide Attempt^a^

	Total (N = 804 617), No. (%)	Admission for suicide attempt at delivery (n = 1202)			Readmission for depression within 1 y post partum (n = 468)			Readmission with suicide attempt within 1 y post partum (n = 111)		
		No. (%)	OR (95% CI)	*P* value	No. (%)	OR (95% CI)	*P* value	No. (%)	OR (95% CI)	*P* value
Admission with suicide attempt at delivery	1202 (0.15)	NA	NA	NA	4 (0.86)	5.78 (2.16-15.49)	.006	7 (6.31)	45.25 (21.00-97.47)	<.001
Readmission to the same hospital within 1 y post partum										
For depression	468 (0.06)	4 (0.33)	5.78 (2.16-15.49)	.006	NA	NA	NA	36 (32.43)	893.42 (593.84-1344.12)	<.001
For suicide attempt	111 (0.01)	7 (0.58)	45.25 (21.00-97.47)	<.001	36 (7.69)	893.42 (593.84-1344.12)	<.001	NA	NA	NA
Nonpsychiatric medical history before delivery										
Current or ex-smoker	69 940 (8.69)	166 (13.81)	1.68 (1.43-1.99)	<.001	69 (14.74)	1.82 (1.41-2.35)	<.001	21 (18.92)	2.45 (1.52-3.94)	<.001
Heavy smoker^b^	562 (0.07)	0	0.00 (NA)	>.99	1 (0.21)	3.07 (0.43-21.86)	.28	2 (1.80)	26.34 (6.49-106.93)	.003
Diabetes	64 239 (7.98)	94 (7.82)	0.98 (0.79-1.21)	.84	48 (10.26)	1.32 (0.98-1.78)	.07	10 (9.01)	1.14 (0.60-2.19)	.69
Hypertension	16 245 (2.02)	23 (1.91)	0.95 (0.63-1.43)	.79	17 (3.63)	1.83 (1.13-2.97)	.01	2 (1.80)	0.89 (0.22-3.61)	>.99
Dyslipidemia	751 (0.09)	1 (0.08)	0.89 (0.13-6.34)	>.99	0	0.00 (NA)	>.99	0	0.00 (NA)	>.99
Graves disease	4096 (0.51)	4 (0.33)	0.65 (0.24-1.74)	.54	0	0.00 (NA)	.18	0	0.00 (NA)	>.99
Hashimoto thyroiditis	13 888 (1.73)	24 (2.00)	1.16 (0.77-1.74)	.47	13 (2.78)	1.63 (0.94-2.83)	.08	1 (1.04)	0.52 (0.07-3.71)	>.99
SLE	1719 (0.21)	0	0.00 (NA)	.19	4 (0.86)	4.03 (1.51-10.80)	.02	0	0.00 (NA)	>.99
Sjögren syndrome	711 (0.09)	1 (0.08)	0.94 (0.13-6.70)	>.99	2 (0.43)	4.86 (1.21-19.54)	.07	0	0.00 (NA)	>.99
RA	1090 (0.14)	2 (0.17)	1.23 (0.31-4.93)	.68	0	0.00 (NA)	>.99	0	0.00 (NA)	>.99
Epilepsy	3851 (0.48)	9 (0.75)	1.57 (0.81-3.03)	.20	12 (2.56)	5.49 (3.09-9.74)	<.001	3 (2.70)	5.78 (1.83-18.21)	.02
No mental conditions	3676 (0.46)	7 (0.58)	1.28 (0.61-2.69)	.51	9 (1.92)	4.28 (2.21-8.29)	<.001	1 (0.90)	1.98 (0.28-14.19)	.40
Multiple sclerosis	204 (0.03)	1 (0.08)	3.29 (0.46-23.52)	.26	0	0.00 (NA)	>.99	0	0.00 (NA)	>.99
Use of oral corticosteroids	11 780 (1.46)	19 (1.58)	1.08 (0.69-1.70)	.74	22 (4.70)	3.32 (2.17-5.10)	<.001	4 (3.60)	2.52 (0.93-6.83)	.08
Obstetric background and procedures										
Elective CD	200 826 (24.96)	272 (22.63)	0.88 (0.77-1.01)	.06	92 (19.66)	0.74 (0.59-0.92)	.008	16 (14.41)	0.51 (0.30-0.86)	.01
Emergency CD	157 540 (19.58)	234 (19.47)	0.99 (0.86-1.51)	.92	104 (22.22)	1.17 (0.94-1.46)	.15	30 (27.03)	1.52 (1.00-2.31)	.048
Vaginal delivery	446 251 (55.46)	696 (57.90)	1.10 (0.99-1.24)	.09	272 (58.12)	1.11 (0.93-1.34)	.25	65 (58.56)	1.13 (0.78-1.66)	.51
Vacuum extraction	26 612 (3.31)	44 (3.66)	1.11 (0.82-1.50)	.49	23 (4.92)	1.51 (0.99-2.30)	.052	1 (0.90)	0.27 (0.04-1.90)	.28
TPL	80 648 (10.02)	103 (8.57)	0.84 (0.69-1.03)	.09	55 (11.75)	1.20 (0.90-1.58)	.21	16 (14.41)	1.51 (0.89-2.57)	.12
Preterm labor	14 105 (1.75)	17 (1.41)	0.80 (0.50-1.30)	.37	13 (2.78)	1.60 (0.92-2.78)	.09	7 (6.31)	3.77 (1.76-8.11)	.004
Threatened miscarriage	4068 (0.51)	3 (0.25)	0.49 (0.16-1.53)	.30	4 (0.86)	1.70 (0.63-4.54)	.31	0	0.00 (NA)	>.99
Miscarriage	4813 (0.60)	5 (0.42)	0.69 (0.29-1.67)	.57	6 (1.28)	2.16 (0.96-4.83)	.06	1 (0.90)	1.51 (0.21-10.82)	.49
Stillbirth	289 (0.04)	0	0.00 (NA)	>.99	0	0.00 (NA)	>.99	0	0.00 (NA)	>.99
Postterm birth	341 (0.04)	1 (0.08)	1.97 (0.28-14.01)	.40	0	0.00 (NA)	>.99	0	0.00 (NA)	>.99
Gestational hypertension	64 863 (8.06)	84 (6.99)	0.86 (0.69-1.07)	.17	57 (12.18)	1.58 (1.20-2.09)	.001	8 (7.21)	0.89 (0.43-1.82)	.86
Lactation mastitis	3855 (0.48)	5 (0.42)	0.87 (0.36-2.09)	>.99	4 (0.86)	1.79 (0.67-4.80)	.29	1 (0.90)	1.89 (0.26-13.53)	.41

Abbreviations: CD, cesarean delivery; NA, not applicable; OR, odds ratio; RA, rheumatoid arthritis; SLE, systemic lupus erythematosus; TPL, threatened preterm labor.

^a^ The prevalence of each potential predisposing risk factor for the occurrence of the evaluated peripartum psychiatric problems among the cumulative 804 617 patients who delivered at hospitals is listed in the column next to the variable names. The following columns list the prevalence of each explanatory variable, stratified by the occurrence of each psychiatric outcome. The *P* value and effect size (unadjusted OR) for the occurrence of each psychiatric outcome are shown next to the prevalence.

^b^ Heavy smokers are those with a Brinkman index of ≥600.

Regarding the evaluated psychiatric outcomes, 1202 patients (0.15%) were hospitalized for suicide attempt and childbirth during the same hospital stay, 468 (0.06%) were readmitted to the same hospital of delivery for depression within 1 year post partum, and 111 (0.01%) were readmitted for suicide attempt within 1 year post partum. Among the 468 women with the second outcome, the readmission occurred a median of 7 weeks (IQR, 2-27 weeks) post partum, with 233 readmissions (49.8%) before 6 weeks and 235 (50.2%) after 6 weeks post partum. Seven patients had both prepartum and postpartum suicide attempts, and 3 of them had prepartum psychiatric problems (1 with 3 psychiatric comorbidities [depression, bipolar disorder, and insomnia], 1 with schizophrenia, and 1 with anxiety disorder).

### Profiles of Patients With Each Psychiatric Outcome

In the 1202 patients with the first outcome (admission with prepartum suicide attempt and delivery during the same hospital stay), the median age was 32 years (IQR, 29-36 years), the median BMI was 24.2 (IQR, 22.1-26.9), and 166 (13.8%) had a history of smoking. None of the patients were heavy smokers. In these 1202 patients, the age distribution was lower (*P* = .02, MWU test), and the rate of smoking history was higher (13.8% vs 8.7%; *P* < .001) compared with the other 803 415 patients without this outcome.

In the 468 patients with the second outcome (readmission for depression within 1 year post partum), the median age was 33 years (IQR, 29-37 years); the median BMI was 24.6 (IQR, 22.0-28.4); and 69 (14.7%) had a history of smoking, including 1 heavy smoker. The age distribution did not differ from the other 804 149 women without this outcome (*P* = .12, MWU test), but the rate of smoking was higher than that among the others (14.7% vs 8.7%; *P* < .001).

In the 111 patients with the third outcome (readmission for suicide attempt within 1 year post partum), the median age was 31 years (IQR, 26-35 years); the median BMI was 25.2 (IQR, 22.2-28.2); and 21 (18.9%) had a history of smoking, including 2 heavy smokers. The age distribution was lower (*P* = .03, MWU test), and the rate of smoking history was higher (18.9% vs 8.7%; *P* < .001) than among the other 804 506 women without this outcome. Readmission for suicide attempt occurred at a median of 22 weeks (IQR, 7-35 weeks) post partum, which was longer than that for depression (*P* < .001, MWU test).

### Associations With Nonpsychiatric Medical History

The prevalence of the evaluated nonpsychiatric medical histories by the presence of each outcome is summarized in Table 1. Epilepsy was associated with postpartum depression (OR, 5.49; 95% CI, 3.09-9.74; *P* < .001). Smoking status was associated with both prepartum (OR, 1.68; 95% CI, 1.43-1.99; *P* < .001) and postpartum (OR, 2.45; 95% CI, 1.52-3.94; *P* < .001) suicide attempts. The prevalences of the evaluated nonpsychiatric histories among patients with prepartum or postpartum suicide attempts did not significantly differ from those among patients without each outcome.

### Associations With Obstetric Factors

The prevalence of obstetric procedures and comorbidities by the presence of each outcome is also summarized in Table 1. Patients with elective CD showed a lower prevalence of postpartum depression (19.66%; *P* = .008) and suicide attempts (14.41%; *P* = .01) vs those without (24.96%), whereas the prevalence of postpartum suicide attempts was slightly higher among women with emergency CD (27.03% vs 19.58% without; *P* = .048). The *P* values for the other evaluated obstetric procedures were not significant.

### Associations With Prenatal Psychiatric Problems

Prevalences of the evaluated psychiatric medical histories and used psychiatric medications by the presence of each outcome are summarized in Table 2. The prevalence of prenatal psychiatric history was lower among patients with prepartum suicide attempts than those with postpartum suicide attempts (4.24% vs 27.93%, *P* < .001).

**Table 2.** Patient Psychiatric Medical History and Administered Drugs at Readmission for Postpartum Depression or Suicide Attempt

	Total (N = 804 617), No. (%)	Admission for suicide attempt at delivery (n = 1202)			Readmission for depression within 1 y post partum (n = 468)			Readmission for suicide attempt within 1 y post partum (n = 111)		
		No. (%)	OR (95% CI)	*P* value	No. (%)	OR (95% CI)	*P* value	No. (%)	OR (95% CI)	*P* value
**Psychiatric medical history before admission for delivery**										
Depression										
Total	2202 (0.27)	24 (2.00)	7.50 (4.99-11.25)	<.001	72 (15.39)	68.46 (53.07-88.32)	<.001	14 (12.61)	52.92 (30.17-92.83)	<.001
Uncomplicated^a^	1238 (0.15)	10 (0.83)	5.48 (2.93-10.24)	<.001	22 (4.70)	32.57 (21.15-50.16)	<.001	3 (2.70)	18.07 (5.73-56.98)	<.001
Schizophrenia										
Total	1262 (0.16)	14 (1.17)	7.57 (4.46-12.87)	<.001	18 (3.85)	25.82 (16.07-41.49)	<.001	11 (9.91)	70.63 (37.80-131.96)	<.001
Uncomplicated^a^	730 (0.09)	6 (0.50)	5.56 (2.49-12.45)	<.001	2 (0.43)	4.74 (1.18-19.03)	.07	5 (4.51)	52.30 (21.26-128.61)	<.001
Alcohol use disorder										
Total	28 (<0.01)	0	0.00 (NA)	>.99	1 (0.21)	63.77 (8.65-470.28)	.02	2 (1.80)	567.73 (133.12-2421.34)	<.001
Uncomplicated^a^	18 (<0.01)	0	0.00 (NA)	>.99	1 (0.21)	101.29 (13.45-762.65)	.01	1 (0.90)	430.21 (56.76-3260.70)	.003
Personality disorder										
Total	388 (0.05)	12 (1.00)	21.54 (12.09-38.37)	<.001	8 (1.71)	36.79 (18.16-74.53)	<.001	5 (4.51)	99.03 (40.16-244.20)	<.001
Uncomplicated^a^	294 (0.04)	8 (0.67)	18.82 (9.30-38.07)	<.001	4 (0.86)	23.90 (8.87-64.37)	<.001	1 (0.90)	24.95 (3.47-179.32)	.04
Insomnia										
Total	3348 (0.42)	13 (1.08)	2.62 (1.52-4.54)	<.001	45 (9.62)	25.79 (18.93-35.14)	<.001	12 (10.81)	29.11 (15.97-53.05)	<.001
Uncomplicated^a^	2400 (0.30)	4 (0.33)	1.12 (0.42-2.98)	.79	8 (1.71)	5.83 (2.89-11.74)	<.001	1 (0.90)	3.04 (0.42-21.78)	.28
Anxiety disorders										
Total	706 (0.09)	2 (0.17)	1.90 (0.47-7.62)	.28	5 (1.07)	12.38 (5.11-29.97)	<.001	6 (5.41)	65.62 (28.73-149.88)	<.001
Uncomplicated^a^	442 (0.06)	1 (0.08)	1.52 (0.21-10.80)	.48	3 (0.64)	11.81 (3.78-36.90)	.002	3 (2.70)	50.88 (16.09-160.84)	<.001
Bipolar disorder										
Total	573 (0.07)	7 (0.58)	8.31 (3.93-17.55)	<.001	13 (2.78)	41.00 (23.48-71.60)	<.001	7 (6.31)	95.60 (44.28-206.43)	<.001
Uncomplicated^a^	236 (0.03)	1 (0.08)	2.85 (0.40-20.30)	.30	2 (0.43)	14.74 (3.66-59.48)	.009	0	0.00 (NA)	>.99
Adjustment disorder										
Total	528 (0.07)	6 (0.50)	7.72 (3.44-17.29)	<.001	5 (1.07)	16.59 (6.85-40.22)	<.001	0	0.00 (NA)	>.99
Uncomplicated^a^	299 (0.04)	2 (0.17)	4.51 (1.12-18.13)	.07	3 (0.64)	17.52 (5.60-54.83)	<.001	0	0.00 (NA)	>.99
Previous suicide attempt	651 (0.08)	29 (2.40)	31.91 (21.89-46.51)	<.001	6 (1.28)	16.18 (7.20-36.33)	<.001	12 (10.81)	152.49 (83.34-278.99)	<.001
**No. of coexisting psychiatric conditions**										
6	2 (<0.01)	0	NA	NA	0	NA	NA	0	NA	NA
5	8 (<0.01)	0	NA	NA	0	NA	NA	0	NA	NA
4	60 (0.01)	1 (0.08)	NA	NA	2 (0.43)	NA	NA	2 (1.80)	NA	NA
3	291 (0.04)	6 (0.50)	NA	NA	14 (2.99)	NA	NA	5 (4.51)	NA	NA
2	1141 (0.14)	12 (1.00)	NA	NA	36 (7.69)	NA	NA	10 (9.01)	NA	NA
1	5721 (0.71)	32 (2.66)	NA	NA	45 (9.62)	NA	NA	14 (12.61)	NA	NA
No mental condition	797 394 (99.10)	1151 (95.76)	0.20 (0.15-0.27)	<.001	371 (79.27)	0.03 (0.03-0.04)	<.001	80 (72.07)	0.02 (0.02-0.04)	<.001
**Used psychiatric drugs**										
Antidepressants (total)	4498 (0.56)	23 (1.91)	NA	NA	82 (17.52)	NA	NA	19 (17.12)	NA	NA
TCA	439 (0.06)	0	NA	NA	3 (0.64)	NA	NA	4 (3.60)	NA	NA
SSRI	2842 (0.35)	15 (1.25)	NA	NA	56 (11.97)	NA	NA	11 (9.91)	NA	NA
SNRI	847 (0.11)	4 (0.33)	NA	NA	21 (4.49)	NA	NA	6 (5.41)	NA	NA
NaSSA	678 (0.08)	8 (0.67)	NA	NA	19 (4.06)	NA	NA	7 (6.31)	NA	NA
Other	662 (0.08)	2 (0.17)	NA	NA	18 (3.85)	NA	NA	4 (3.60)	NA	NA
Benzodiazepines	8488 (1.06)	34 (2.83)	NA	NA	101 (21.58)	NA	NA	30 (27.03)	NA	NA
Z-drugs^b^	11 585 (1.44)	35 (2.91)	NA	NA	62 (13.25)	NA	NA	20 (18.02)	NA	NA

Abbreviations: NA, not applicable; NaSSA, noradrenergic and specific serotonergic antidepressant; OR, odds ratio; SNRI, serotonin-norepinephrine reuptake inhibitor; SSRI, selective serotonin reuptake inhibitor; TCA, tricyclic antidepressant; Z-drug, nonbenzodiazepine γ-aminobutyric acid receptor agonist.

^a^ Cases without other coexisting psychiatric conditions.

^b^ Zolpidem, zopiclone, and eszopiclone.

Regarding the occurrence of the first outcome, uncomplicated histories of personality disorder (OR, 18.82; 95% CI, 9.30-38.07; *P* < .001), schizophrenia (OR, 5.56; 95% CI, 2.49-12.45; *P* < .001), and depression (OR, 5.48; 95% CI, 2.93-10.24; *P* < .001) were potential risk factors. Prenatal history of suicide attempt (OR, 31.91; 95% CI, 21.89-46.51; *P* < .001) was also a significant risk factor. For the occurrence of the second outcome, uncomplicated histories of alcohol use disorder (OR, 101.29; 95% CI, 13.45-762.65; *P* = .01), depression (OR, 32.57; 95% CI, 21.15-50.16; *P* < .001), personality disorder (OR, 23.90; 95% CI, 8.87-64.37; *P* < .001), adjustment disorder (OR, 17.52; 95% CI, 5.60-54.83; *P* < .001), bipolar disorder (OR, 14.74; 95% CI, 3.66-59.48; *P* = .009), anxiety disorders (OR, 11.81; 95% CI, 3.78-36.90; *P* = .002), and insomnia (OR, 5.83; 95% CI, 2.89-11.74; *P* < .001) were potential risk factors. Prenatal history of suicide attempt (OR, 16.18; 95% CI, 7.20-36.33; *P* < .001) was also a significant potential risk factor. For the occurrence of the third outcome, alcohol use disorder (OR, 430.21; 95% CI, 56.76-3260.70; *P* = .003), schizophrenia (OR, 52.30; 95% CI, 21.26-128.61; *P* < .001), anxiety disorders (OR, 50.88; 95% CI, 16.09-160.84; *P* < .001), personality disorder (OR, 24.95; 95% CI, 3.47-179.32; *P* = .04), and depression (OR, 18.07; 95% CI, 5.73-56.98; *P* < .001) were potential risk factors. Prenatal history of suicide attempt (OR, 152.49; 95% CI, 83.34-278.99; *P* < .001) was also a significant potential risk factor. Regarding the used sedative-hypnotics, patients taking benzodiazepines showed higher frequencies of readmission for depression (1.19% vs 0.54%; *P* < .001) or suicide attempt (0.35% vs 0.17%; *P* = .01) than those taking Z-drugs.

### Multivariable Binary Logistic Regression

Multivariable regression analysis of the overall patient population was performed for each outcome by combining all eligible explanatory variables into each regression model (Table 3). In all models, the variance inflation factor values for the enrolled potential risk factors were smaller than 1.5.

**Table 3.** Multivariable Binary Logistic Regression Analysis of the Entire Cohort^a^

Factor	Adjusted OR (95% CI)	*P* value	VIF
Hospitalization with suicide attempt and delivery during the same hospital stay			
(Constant)	NA	<.001	NA
Age	0.99 (0.98-1.00)	.008	1.000
Schizophrenia	2.89 (1.52-5.50)	.001	1.191
Depression	3.97 (2.35-6.70)	<.001	1.244
Insomnia	1.01 (0.54-1.91)	.98	1.125
Personality disorder	10.81 (5.70-20.49)	<.001	1.075
Bipolar disorder	1.94 (0.81-4.62)	.14	1.357
Adjustment disorder	2.66 (1.07-6.58)	.03	1.147
Readmission for depression within 1 y post partum			
(Constant)	NA	<.001	NA
Age	1.01 (1.00-1.03)	.13	1.000
Schizophrenia	1.57 (0.89-2.78)	.12	1.198
Depression	34.01 (23.71-48.79)	<.001	1.250
Insomnia	3.73 (2.44-5.69)	<.001	1.140
Anxiety disorders	1.05 (0.41-2.71)	.91	1.132
Alcohol use disorder	7.77 (0.89-68.28)	.06	1.075
Personality disorder	5.00 (2.24-11.16)	<.001	1.078
Bipolar disorder	1.36 (0.72-2.56)	.34	1.358
Adjustment disorder	1.04 (0.40-2.71)	.94	1.152
Readmission for suicide attempt within 1 y post partum			
(Constant)	NA	.07	NA
Age	0.96 (0.93-1.00)	.03	1.000
Schizophrenia	5.77 (2.17-15.38)	<.001	1.195
Depression	7.27 (2.95-17.91)	<.001	1.238
Insomnia	3.17 (1.30-7.78)	.01	1.133
Anxiety disorders	8.13 (2.88-22.98)	<.001	1.129
Alcohol use disorder	163.54 (28.30-944.95)	<.001	1.073
Heavy smoker	23.09 (5.46-97.62)	<.001	1.030
Personality disorder	10.28 (3.29-32.10)	<.001	1.082
Bipolar disorder	3.98 (1.36-11.67)	.01	1.359

Abbreviations: NA, not applicable; OR, odds ratio; VIF, variance inflation factor.

^a^ Overall, there were 804 617 participants. In the respective regression models, all eligible explanatory variables were simultaneously entered into the model to be controlled. Multicollinearity of the risk factors were evaluated by calculating the VIF for each variable.

Regarding the first outcome, a prenatal history of personality disorder (adjusted OR [aOR], 10.81; 95% CI, 5.70-20.49; *P* < .001), depression (aOR, 3.97; 95% CI, 2.35-6.70; *P* < .001), schizophrenia (aOR, 2.89; 95% CI, 1.52-5.50; *P* = .001), adjustment disorder (aOR, 2.66; 95% CI, 1.07-6.58; *P* = .03), and younger age (aOR per 1 year, 0.99; 95% CI, 0.98-1.00; *P* = .008) were significant risk factors. Regarding the second outcome, prenatal histories of depression (aOR, 34.01; 95% CI, 23.71-48.79; *P* < .001), personality disorder (5.00; 2.24-11.16; *P* < .001) and insomnia (aOR, 3.73; 95% CI, 2.44-5.69; *P* < .001) were significant risk factors. Regarding the third outcome, prenatal histories of alcohol use disorder (aOR, 163.54; 95% CI, 28.30-944.95; *P* < .001), heavy tobacco use (aOR, 23.09; 95% CI, 5.46-97.62; *P* < .001), personality disorder (aOR, 10.28; 95% CI, 3.29-32.10; *P* < .001), anxiety disorders (aOR, 8.13; 95% CI, 2.88-22.98; *P* < .001), depression (aOR, 7.27; 95% CI, 2.95-17.91; *P* < .001), schizophrenia (aOR, 5.77; 95% CI, 2.17-15.38; *P* < .001), bipolar disorder (aOR, 3.98; 95% CI, 1.36-11.67; *P* = .01), insomnia (aOR, 3.17; 95% CI, 1.30-7.78; *P* = .01), and younger age (aOR per 1 year, 0.96; 95% CI, 0.93-1.00; *P* = .03) were significant risk factors.

To check the robustness of the observed statistical significance from the multivariable analyses, sensitivity analyses were performed for each model after excluding patients with a prenatal history of each outcome. After excluding patients with prenatal suicide attempt, there were 1173 admissions with the first outcome and 99 readmissions with the third outcome. After excluding patients with prenatal depression, there were 396 readmissions with the second outcome. The results of the sensitivity analysis are shown in Table 4. Regarding the first outcome, the significance in prenatal adjustment disorder (aOR, 2.82; 95% CI, 0.99-8.02; *P* = .052) disappeared. Regarding the second outcome, prenatal histories of adjustment disorder (aOR, 10.28; 95% CI, 3.64-29.08; *P* < .001), bipolar disorder (aOR, 8.11; 95% CI, 2.13-30.87; *P* = .002), and anxiety disorders (aOR, 3.78; 95% CI, 1.11-12.80; *P* = .03) remained significant. Regarding the third outcome, the significance in prenatal personality disorder (aOR, 4.42; 95% CI, 0.56-35.16; *P* = .16) disappeared.

**Table 4.** Sensitivity Analysis for the Multivariable Logistic Regression After Excluding Patients With a Prepartum History of the Focused Outcome^a^

Factor	Adjusted OR (95% CI)	*P* value	VIF
Hospitalization for suicide attempt and childbirth (after excluding patients with prepartum suicide attempt; n = 803 966)			
Constant	NA	<.001	NA
Age	0.99 (0.98-1.00)	.009	1.000
Schizophrenia	3.20 (1.61-6.36)	<.001	1.175
Depression	2.61 (1.40-4.89)	.003	1.238
Insomnia	1.16 (0.60-2.25)	.66	1.120
Personality disorder	12.44 (6.38-24.23)	<.001	1.059
Bipolar disorder	2.11 (0.78-5.70)	.14	1.349
Adjustment disorder	2.82 (0.99-8.02)	.052	1.126
Readmission for depression within 1 y post partum (after excluding those with prepartum depression; n = 802 415)			
Constant	NA	.03	NA
Age	1.02 (1.00-1.04)	.08	1.000
Schizophrenia	0.97 (0.21-4.39)	.97	1.117
Insomnia	4.14 (2.00-8.56)	<.001	1.070
Anxiety disorder	3.78 (1.11-12.80)	.03	1.099
Alcohol use disorder	13.33 (0.63-284.32)	.10	1.061
Personality disorder	13.81 (4.51-42.26)	<.001	1.046
Bipolar disorder	8.11 (2.13-30.87)	.002	1.083
Adjustment disorder	10.28 (3.64-29.08)	<.001	1.106
Readmission for suicide attempt within 1 y post partum (after excluding those with prepartum suicide attempt; n = 803 966)			
Constant	NA	.43	NA
Age	0.96 (0.93-1.00)	.03	1.000
Schizophrenia	11.66 (4.00-33.95)	<.001	1.181
Depression	3.55 (1.17-10.83)	.03	1.235
Insomnia	3.15 (1.11-8.96)	.03	1.128
Anxiety disorder	8.81 (2.77-28.02)	<.001	1.121
Alcohol use disorder	183.66 (22.85-1476.33)	<.001	1.070
Heavy smoker	12.05 (1.62-89.81)	.02	1.030
Personality disorder	4.42 (0.56-35.16)	.16	1.064
Bipolar disorder	3.81 (1.03-14.14)	.05	1.350

Abbreviations: NA, not applicable; OR, odds ratio; VIF, variance inflation factor.

^a^ Sensitivity analyses to check for the reproducibility of the multivariable binary logistic regression analysis in the overall cohort of eligible patients were performed, after excluding patients with a prepartum history of the focused psychiatric outcome (suicide attempt or depression). In the respective regression models, all eligible explanatory variables were simultaneously entered into the model to be controlled.

## Discussion

This cohort study used retrospective data to identify prenatal psychiatric histories, including personality disorder, anxiety disorders, depression, and schizophrenia, as potential independent risk factors for postpartum suicide attempts. In addition to these psychiatric histories, younger age and heavy alcohol and tobacco consumption were found to be significant risk factors. A prenatal history of epilepsy was a potential risk factor for postnatal depression, although whether this finding was caused by the disease itself or the use of antiepileptic drugs is uncertain.^26^ Our results indicate the importance of paying additional attention to pregnant women with miscellaneous prenatal psychiatric problems other than depression. In the general population, schizophrenia,^27^ personality disorder,^28^ alcohol use disorder,^29,30^ anxiety disorders,^31^ bipolar disorder,^32^ adjustment disorder,^33^ and insomnia^34^ are reportedly independent risk factors for subsequent suicidal behaviors. Our study found that these risk factors are also significant among pregnant women. Another notable finding of this study was that 1202 patients (0.15%) were hospitalized with a prepartum suicide attempt and delivered their children during the same hospital stay. Pregnant women are known to be more likely to endorse suicidal ideation than the general population.^35^ Although our data did not indicate how violent and serious these suicidal behaviors were, this finding implies the importance of paying close attention to women with predisposing risk factors, even before childbirth. In particular, we found that women with previous suicidal behaviors are at least 10 times more likely to attempt suicide peripartum. A meta-analysis showed that active contact and follow-up interventions are effective at preventing a repeated suicide attempt among patients with previous suicide attempts.^36^ Implementing brief psychological interventions, such as a volitional help sheet, may reduce the risk of repeating suicidal behaviors.^37,38,39^ In addition to these psychotherapeutic interventions, social support from family and friends may also reduce the risk of future attempts.^40^ The evidence for the effectiveness of antipsychotic agents in preventing a repeated suicide attempt is currently limited, necessitating a thoughtful consideration in prescribing antidepressants during pregnancy.^41,42,43^ Our findings reveal a higher incidence of peripartum suicide attempts among patients who take benzodiazepine hypnotics than among those taking Z-drugs. However, the 2 populations may have different characteristics, and the suicide risk could be inherent to the different profiles. The safety of Z-drugs during pregnancy is yet to be fully established, and careful consideration is needed in prescribing these drugs to pregnant women.

### Limitations

This study has several limitations. First, the data regarding the times of delivery (parity), detailed maternal personality profile, negative life events, marital status, intimate partner violence, education level, income situation, social support level, and paternal factors were not available. As some of these variables are possible risks for peripartum mental health problems,^44,45,46,47,48,49^ patients with these unevaluated risks would require additional attention. Second, this study only evaluated the women who delivered at hospitals that joined the DPC system. Women who delivered in other places or whose medical costs were completely covered by self-pay were not analyzed. Based on the statistics released by the Japanese government, 47.1% of childbirths in the country took place in nonhospital settings in 2021.^50^ Third, we could not identify women who were admitted for depression or attempted suicide at different hospitals from where they had delivered. Peripartum women who completed suicide and were not admitted to the hospital also could be missed; therefore, this study may underestimate the incidence of peripartum suicide attempt, and risk estimations could be biased toward the null. Fourth, the observed incidence of prepartum (0.15%) and postpartum (0.01%) suicide attempts appeared to be lower than expected from previous reports. A meta-analysis estimated the pooled prevalence of suicide attempts in 0.10% to 4.69% of pregnant women and 0.01% to 3.21% in postpartum women.^51^ This discrepancy could have been derived from the design of this study. Fifth, although data on race and ethnicity were not collected in this study, our cohort is considered to mainly comprise individuals of Asian ancestry. Asian populations have been shown to have a lower lifetime prevalence of mental disorders compared with other racial groups.^52^ Racial, ethnic, environmental, and cultural factors are interactively associated with regional variation in mental disorders.^53^ Similar studies in other countries are needed to confirm the generalizability of our findings.

## Conclusions

In this cohort study, we found that younger age; heavy alcohol and tobacco consumption; and a prenatal history of suicide attempt, personality disorder, anxiety disorders, depression, and schizophrenia are potential risk factors for postpartum suicide attempts. Postpartum women with these risks may require additional caution and interventions to avoid fatal maternal events.

## Notes

Supplement.
Data Sharing Statement
