# Supplementary material for: Risk Factors Associated With Peripartum Suicide Attempts in Japan
Source: JAMA Netw Open. 2023 Jan 12;6(1):e2250661. doi: 10.1001/jamanetworkopen.2022.50661 (PMC9857025; doi:10.1001/jamanetworkopen.2022.50661)
Supplement: Supplement. — Data Sharing Statement [file jamanetwopen-e2250661-s001.pdf]

Data Sharing Statement

Akaishi. Risk Factors Associated With Peripartum Suicide Attempts in Japan. *JAMA Netw Open*. Published January 12, 2023. doi:10.1001/jamanetworkopen.2022.50661

Data
Data available: Yes
Data types: Deidentified participant data
How to access data: The anonymized data that supprot the findings of this study are available from the corresponding author.
When available: With publication

Supporting Documents
Document types: None

Additional Information
Who can access the data: The data will be made available to researchers whose proposed use of the data has been approved.
Types of analyses: For research purposes or for reproducing the results.
Mechanisms of data availability: After approval of a proposal.
Any additional restrictions: Under the protection of the Personal Information Law and the Ethical Guidelines for Medical and Health Research Involving Human Subjects in Japan.
